# Leaf wettability and leaf angle affect air-moisture deposition in wheat for self-irrigation

**DOI:** 10.1186/s12870-023-04123-z

**Published:** 2023-02-27

**Authors:** Sadia Hakeem, Zulfiqar Ali, Muhammad Abu Bakar Saddique, Sabah Merrium, Muhammad Arslan, Muhammad Habib-ur-Rahman

**Affiliations:** 1 Institute of Plant Breeding and Biotechnology, MNS University of Agriculture, Multan, Pakistan; 2 Department of Plant Breeding and Genetics, University of Agriculture, Faisalabad, Pakistan; 3 Programs and Projects Department, Islamic Organization for Food Security, Mangilik Yel Ave. 55/21 AIFC, Unit 4, C4.2, Astana, Kazakhstan; 4 Institute of Crop Science and Resource Conservation (INRES), Crop Science Group, University of Bonn, Bonn, Germany; 5 Department of Agronomy, MNS University of Agriculture, Multan, Pakistan

**Keywords:** Climate change, Contact angle, Hydrophilic, Leaf rolling, Leaf angle, Precise irrigation

## Abstract

**Background:**

Climate change and depleting water sources demand scarce natural water supplies like air moisture to be used as an irrigation water source. Wheat production is threatened by the climate variability and extremes climate events especially heat waves and drought. The present study focused to develop the wheat plant for self-irrigation through optimizing leaf architecture and surface properties for precise irrigation.

**Methods:**

Thirty-four genotypes were selected from 1796 genotypes with all combinations of leaf angle and leaf rolling. These genotypes were characterized for morpho-physiological traits and soil moisture content at stem-elongation and booting stages. Further, a core set of ten genotypes was evaluated for stem flow efficiency and leaf wettability.

**Results:**

Biplot, heat map, and correlation analysis indicated wide diversity and traits association. The environmental parameters indicated substantial amount of air moisture (> 60% relative humidity) at the critical wheat growth stages. Leaf angle showed negative association with leaf rolling, physiological and yield traits, adaxial and abaxial contact angle while leaf angle showed positive association with the stem flow water. The wettability and air moisture harvesting indicated that the genotypes (coded as 1, 7, and 18) having semi-erect to erect leaf angle, spiral rolling, and hydrophilic leaf surface (<90^o^) with contact angle hysteresis less than 10^o^ had higher soil moisture content (6-8%) and moisture harvesting efficiency (3.5 ml).

**Conclusions:**

These findings can provide the basis to develop self-irrigating, drought-tolerant wheat cultivars as an adaptation to climate change.

**Supplementary Information:**

The online version contains supplementary material available at 10.1186/s12870-023-04123-z.

## Background

Wheat is a staple cereal of around 40% of the world population [1]. With the estimated 9 billion world population by 2050, the wheat yield demand would increase by 60% as compared to 2010. To fulfill this demand, the wheat yield should increase at a steady rate of 1.5% per year (estimation made from 2011 to 2050) [2]. Studies indicate an increase in irrigation requirements reaching up to 74% in worst scenarios for the wheat crop due to increased evapotranspiration owing to the rise in temperature [3, 4]. Under this scenario, better exploitation of available water sources is inevitable for sustainable wheat yield.

The climate change and natural habitats of semi-arid environments have inspired many studies of plant leaf surface structures [5, 6]. These studies have led to the discovery of several novel surface structures like leaf pubescence/trichomes in *Salsola crassa* [7, 8], leaf rolling, leaf to stem angle in *Stipagrostis sabulicola* [9] and super-hydrophobicity of *Nelumbo nucifera* leaves (Lotus) [10] and water channeling in *Phyllostachys aurea* [6]. The advancements in the understanding of these surface traits have led to a quest for similar structures in economically important crops and their replication for technological applications [11]. Plant surfaces have displayed a wide variation for the leaf surface wettability being super-hydrophilic to super-hydrophobic. Studies indicated different wettability characteristics on either surface of the leaf and even dual wettability patterns on different patches of the same leaf [6]. Mostly, these surface structures are related to the local harsh environmental conditions like in Namib grass *Stipagrostis sabulicola* [9] that has fascinating leaf architecture to capture the water from air mist instead of soil. Moreover, it can store the water in its root zone [12]. Despite the extensive studies on the plant surface structure about wettability, there are still limited reports of leaf wettability and leaf angle in crop plants for air moisture capturing and channeling.

In Pakistan, wheat is grown in November and plenty of fog events occur during its growing season. Soil moisture, Indus River, and Western disturbances are the sources of air moisture and fog formation in winter. The plains of Punjab and upper Sindh have shown a tremendous increase in dense fog events in recent decades due to aerosols [13]. The erratic rainfalls and decreased precipitation has been observed for the region while future prediction scenario indicates increasing variability in this trend [14]. A 30-year climate data indicates an increase in the average daily temperature (Fig. S1). Inspired from the Namib dune grass *Stipagrostis sabulicola* [12], the wheat germplasm was explored for the leaf angle (erect to droopy) and surface wettability (hydrophilic to hydrophobic on adaxial and abaxial leaf surface) like in *Dracaena draco* [15]. Literature indicates the differential leaf angle in wheat throughout the canopy [16] can be successfully engineered for the water droplet movement during the moisture harvesting mechanism. Similar studies can also be found for leaf wettability in wheat that showed variability in relation to the wax chemistry and glaucousness of the leaf [17]. Although, a few studies have been reported recently for air moisture harvesting by utilizing leaf angle and wettable leaf surface traits in wheat for self-irrigation [18, 19]. Still, there is a need to explore similar traits in wheat plant for self-irrigation purpose. The present study focused on efficient air moisture harvesting during fog events by the wheat plant through stem flow. The aim was to test the hypothesis that (1) leaf hydrophilicity increases the leaf surface wettability and droplet coalition in wheat (2) erect leaf to stem angle enhances the soil moisture through stem flow (3) leaf angle can be associated with the yield traits and surface wettability to enhance yield. This mechanism can be engineered in wheat and other cereal crops for self-irrigation as a strategy of adaptation to climate change.

## Material and methods

### Site description and experimental design

Thirty-four genotypes including four check cultivars with all possible combinations of leaf angle (droopy, semi-droopy, semi-erect, and erect) and leaf rolling (abaxially, adaxially, and spirally rolled) were selected from diverse germplasm of 1796 genotypes (unpublished data, Fig. S2, Table S1) provided by the Institute of Plant Breeding and Biotechnology, MNS- University of Agriculture, Multan. Long-term climate data (1984-2013) of Multan (30° 12′ N, 71° 26′ E, 122 m elevation) were obtained from the Pakistan Meteorological Department (PMD) to estimate historical climatic patterns (Fig. S1). The weather variables including solar radiation (MJ m^− 2^), wind direction (^o^), wind speed (m s^− 1^), relative humidity (%), rainfall (mm), soil and air temperature (°C), leaf wetness and dryness (minutes) at an interval of 15 and 60 min were observed using an automatic weather station (Campbell Scientific Inc., Logan, UT, USA) installed in the vicinity of experiment. The experimental material was sown following a randomized incomplete block design with three replications in 102 plots in the experimental field of MNS-University of Agriculture, Multan during the growing season of 2018-19. The genotypes were selected based on the leaf rolling pattern, leaf-stem angle, physiological and yield traits. These genotypes were grown in a similar experimental design in 2019-20 under the same field conditions. Row to row distance was maintained at 16 cm and the harvested plot area was five m^2^. The di-ammonium phosphate and sulfate of potash were applied during soil preparation at the rate of 50 kg ha^− 1^. Recommended agronomic management practices for wheat in this region were applied [20].

### Morpho-physiological characterization

The germplasm was characterized for leaf angle and leaf rolling at the stem elongation (33 Zadok’s code), early booting stages (41 Zadok’s code) and anthesis stage (62 Zadok’s code) [21]. The angle between the leaf blade midrib to stem was measured for the first leaf (except flag leaf) of the plant using a protector. The observed leaf angle was categorized into four categories including erect, semi-erect, semi-droopy, and droopy. The leaf rolling was recorded on a quantitative basis following the scale from A Pask, J Pietragalla, D Mullan and M Reynolds [22]. The phenotype was categorized as inward (adaxial leaf surface rolls upward), inward-outward (spiral curling), and outward (abaxial leaf surface facing away from the stem). Other morphological traits including flag leaf attitude, flag leaf twist, plant height, days to heading and maturity, peduncle length, and yield parameters such as spike length, spike weight, spikelet per spike, and seeds per spike and grain yield per plot were recorded at appropriate growth stages of the crop.

Physiological traits including net photosynthetic rate (Pn) (μmol CO_2_ m^− 2^ s^− 1^), stomatal conductance (gs) (mmol H_2_O m^− 2^ s^− 1^), photosynthetic water use efficiency (WUE) (mmol CO_2_ mol^− 1^ H_2_O), and transpiration rate (E) (mmol H_2_O m^− 2^ s^− 1^) were also recorded at the 33 and 41 Zadok’s growth stages. These traits were recorded using the portable photosynthesis system (CIRAS-3, PP Systems, Amesbury, USA) during mid-day (10:00 am and 2:00 pm) under ambient conditions. The expanded portion of the leaf blade of the first leaf was used for data recording. Three readings were recorded for each genotype and an average was calculated.

### Measurement of stem flow and leaf wettability

The air moisture deposited on leaves that finally reached the ground through stem flow was quantified using a collector apparatus. The collector was designed as mentioned by S Hakeem, Z Ali, MAB Saddique, M Habib-ur-Rahman and R Trethowan [23]. The apparatus was set up on three plants for each genotype in the evening (4:00 pm), left overnight, and sampled the next day in the early morning (09:00 am). The actual amount of stem flow water was calculated by subtracting the moisture absorbed by the control collector to exclude the absorption effects of cotton itself. To measure the soil moisture content for comparison, a plant with a similar canopy was used as a control. Soil moisture was measured using a portable soil moisture meter TDR 350 (Spectrum Technologies, United Kingdom). Soil moisture was also measured through the gravimetric method for further validation.

To measure the leaf surface wettability, static contact angle (SCA) of the abaxial and adaxial leaf surface was measured by depositing a droplet of three μl volume at the dosing rate of 0.5 μl/s using the contact angle device (OCA 25, data-physics instruments GmbH, Germany). Measurements were taken at room temperature (16-18 °C, 67-71% relative humidity). Five readings were taken for each genotype and an average was calculated. Based on the SCA, a diverse group of seven genotypes was selected for the contact angle hysteresis (CAH) following the sessile-drop goniometry method (Huhtamäki et al., 2018). The volume of the drop was gradually increased at a dosing rate of 0.05 μl /s from 3 to 8 μl /s for advancing contact angle (ACA) and vice versa for receding contact angle (RCA). Average was calculated from five measurements.

### Statistical analysis

The frequency distributions graphs were plotted using Sigma Plot 14.0. The analysis of variance and Pearson correlation analysis was performed using R version 3.6.1. The leaf traits data, physiological and moisture traits at the 33 and the 41 stages were analyzed using genotype-trait biplot analysis (R version 3.6.1) [24]. The desired leaf traits were evaluated at the lower scale values (e.g., erect leaf angle and inward rolling at scale 1). For the screening of genotypes based on the combination of all the traits under study, a heat map based on clustering was developed using TBtools.

## Results

### Climate indicators

**Fig. 1:**
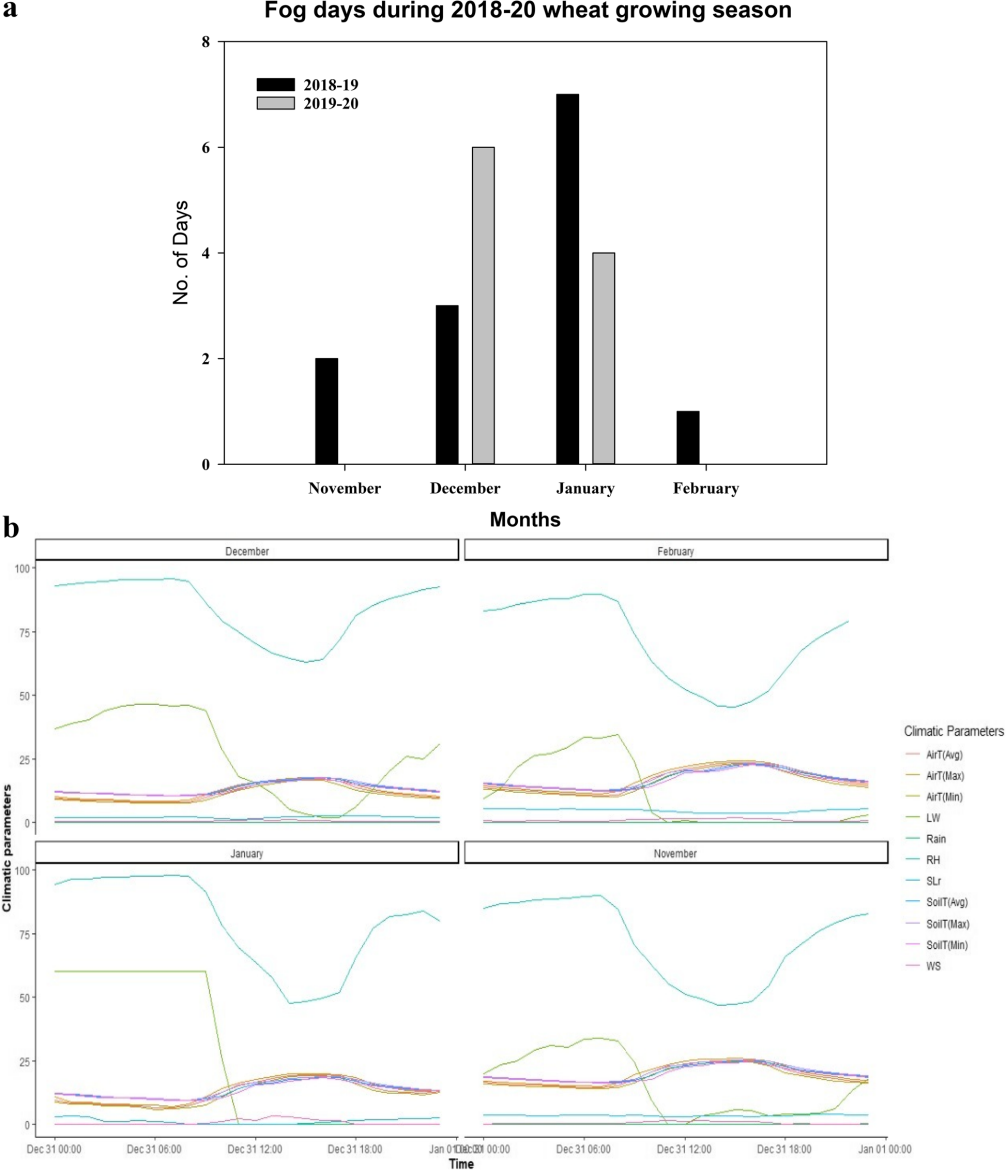
Climate condition during the wheat growing season 2018-19 and 2019-20. **a** Number of fog days in 2018-19 and 2019-20. **b** Hourly weather variables including maximum, minimum, average soil and air temperature, solar radiation, rainfall, relative humidity, windspeed, and leaf wetness minutes. RH: relative humidity (%), SLr: solar radiation (MJm^− 2^), AirT_max: Maximum air temperature (°C), AirT_min: Minimum air temperature (°C), AirT_avg: Average air temperature (°C); SoilT_max: Maximum Soil temperature (°C), SoilT_min: Minimum soil temperature (°C), SoilT_avg: Average soil temperature (°C); LW: Leaf wetness (Minutes); Rain: Rainfall (mm); WS: Wind speed

The hourly weather data indicated a total of thirteen and ten fog days during November to February in 2018-19 and 2019-20, respectively (Fig. 1a). However, in former, maximum fog days (seven) were observed in January while in later, these were recorded in December. Medium to shallow fog persisted in the Multan region [13]. The maximum leaf wetness hours were observed during 00:00 hrs. to 8:00 hrs. experiencing a sudden decline between 09:00 to 18:00 hrs. and then started to rise again (Fig. 1b). The soil temperature, air temperature and wind speed showed inverse relationship, with temperature being maximum during mid-day hours.

### Leaf-traits dynamics

**Fig. 2:**
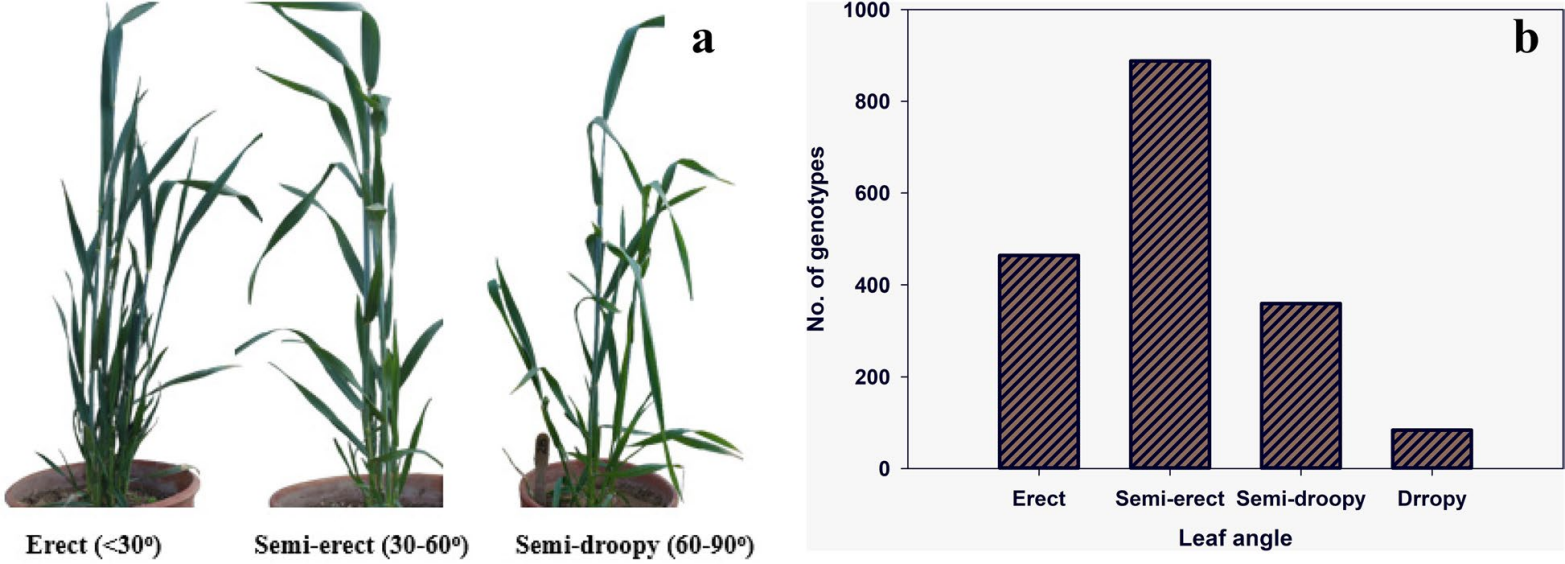
The dynamics of leaf angle across 34 wheat genotypes. **a** Photographs of wheat genotypes categorized according to their leaf-stem angle (photographs of these genotypes under field conditions are presented in Fig. S3). **b** Frequency distribution of the leaf angle for 34 wheat genotypes at the stem elongation and booting stages

The analysis of variance indicated significant differences among genotypes for all the traits under study (Tables S2 and S3). The genotypes were categorized as erect (less than 30^o^), semi-erect (30-60^o^), semi-droopy (60-90^o^), and droopy (more than 90^o^) (Fig. 2a). Among 34 genotypes, none of the genotypes showed droopy leaf angle while most genotypes showed semi-erect leaf angle (22 and 25 at the stem-elongation and booting stage, respectively) while only three and two genotypes showed erect angle. The genotypes having semi-droopy angle were nine and seven at both the stages, respectively (Fig. 2b).

**Fig. 3:**
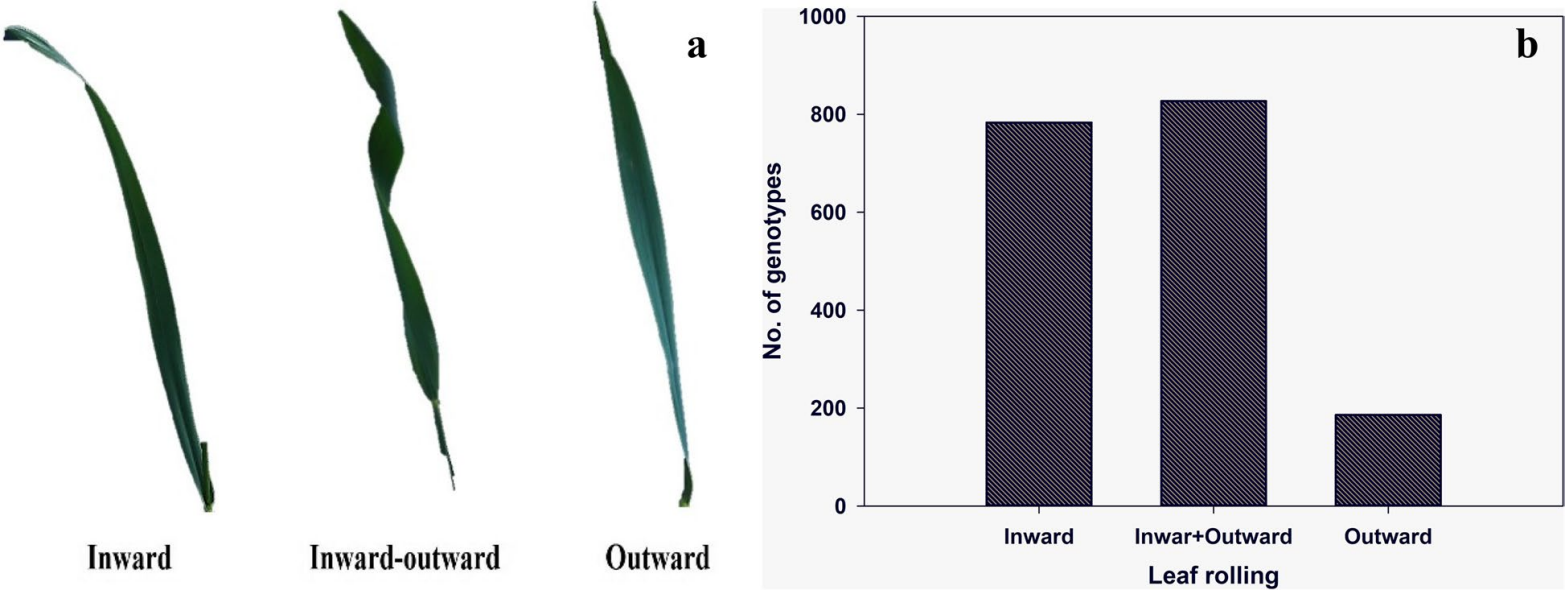
The dynamics of leaf rolling across 34 wheat genotypes. **a** Photographs of wheat leaves showing distinct kinds of rolling. **b** Frequency distribution of the leaf rolling for 34 wheat genotypes at the stem elongation and booting stages

Similarly, genotypes also showed variation for the leaf rolling as presented in Fig. 3a. Maximum genotypes (29) showed inward rolling at stage 41 while only 13 had inward leaf rolling at stage 33. The number of genotypes showing spiral (inward-outward) leaf rolling was 17 and four at the stem elongation and booting stages, respectively. While only four and one genotypes showed outward leaf rolling at both stages, respectively (Fig. 3b).

**Fig. 4:**
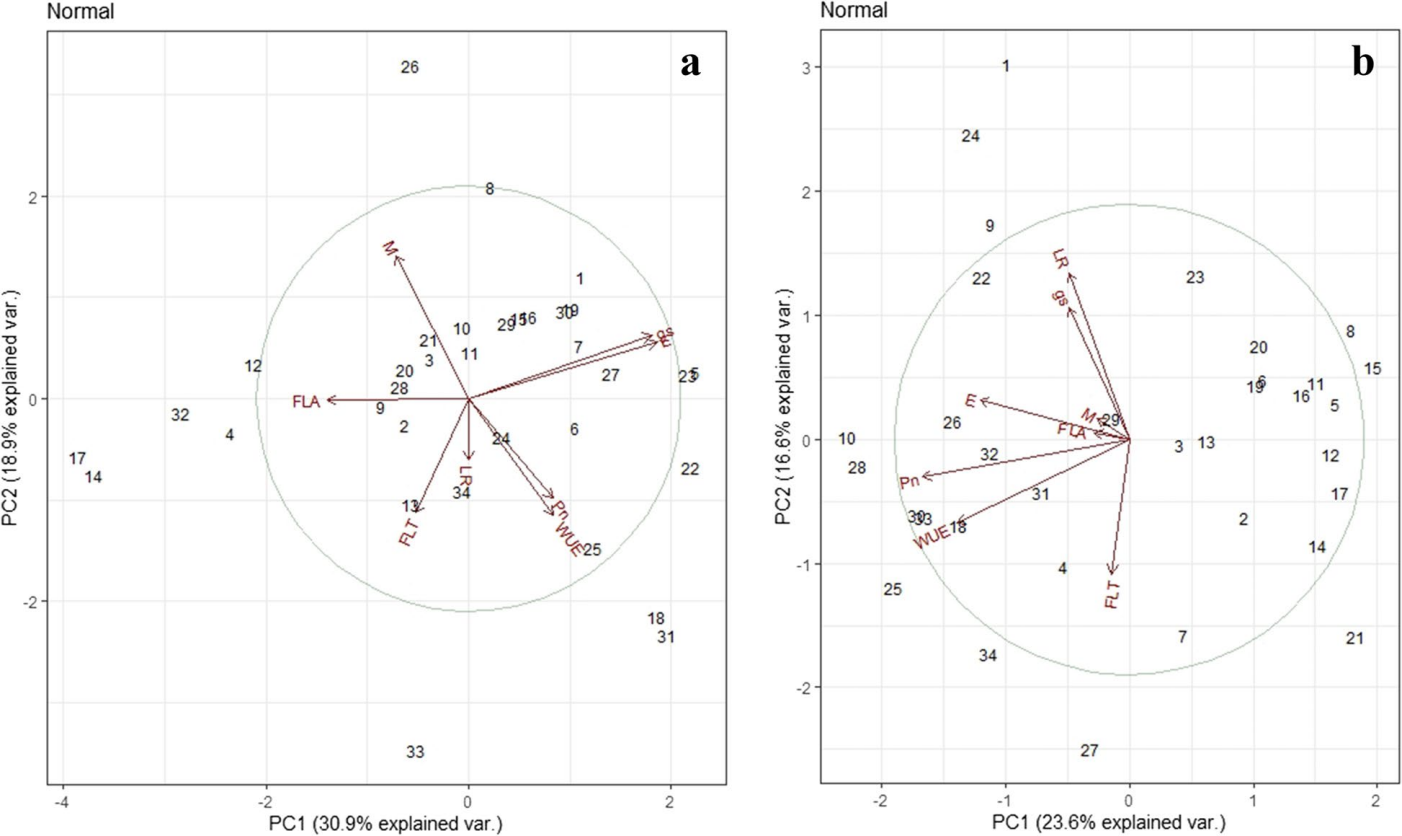
Biplot analysis of 34 wheat genotypes for leaf angle, leaf rolling, soil moisture content, and physiological parameters at the stem elongation stage (**a**) and the booting stage (**b**). LA: leaf-stem angle, LR: leaf rolling; M: Difference of soil moisture content in the root zone and vicinity, E: Transpiration, Pn: Photosynthesis, gs: Stomatal conductance, WUE: Photosynthetic water use efficiency; FLA: flag leaf attitude; FLT: flag leaf traits. The circle indicates the default confidence interval of 68% explaining the theoretical maximum extent of the arrows. Arrows show the correlation among the traits

To evaluate the performance of genotypes for the leaf traits, soil moisture difference, and physiological traits at the stem elongation and booting stages, genotype-traits biplot analysis was performed (Fig. 4a and b, respectively). Genotype 7 showed the highest score for soil moisture content at both the stages and erect leaf-stem angle. Genotype 23 (at stem elongation stage) and genotype 10, and 24 (at booting stage) showed the highest values for E (1.41 and 3.17 mmol H_2_O m^− 2^ s^− 1^, respectively) and gs (96 and 156 mmol H_2_O m^− 2^ s^− 1^, respectively). However, genotypes 18 and 22 managed to maintain higher plot yield (312 g/m^2^) due to the high rate of photosynthesis (9-10 μmol CO_2_ m^− 2^ s^− 1^). The trend of genotypes at the anthesis stage for leaf traits and physiological traits was similar to the booting stage (Fig. S4).

### Association among morpho-physiological and yield traits

**Fig. 5:**
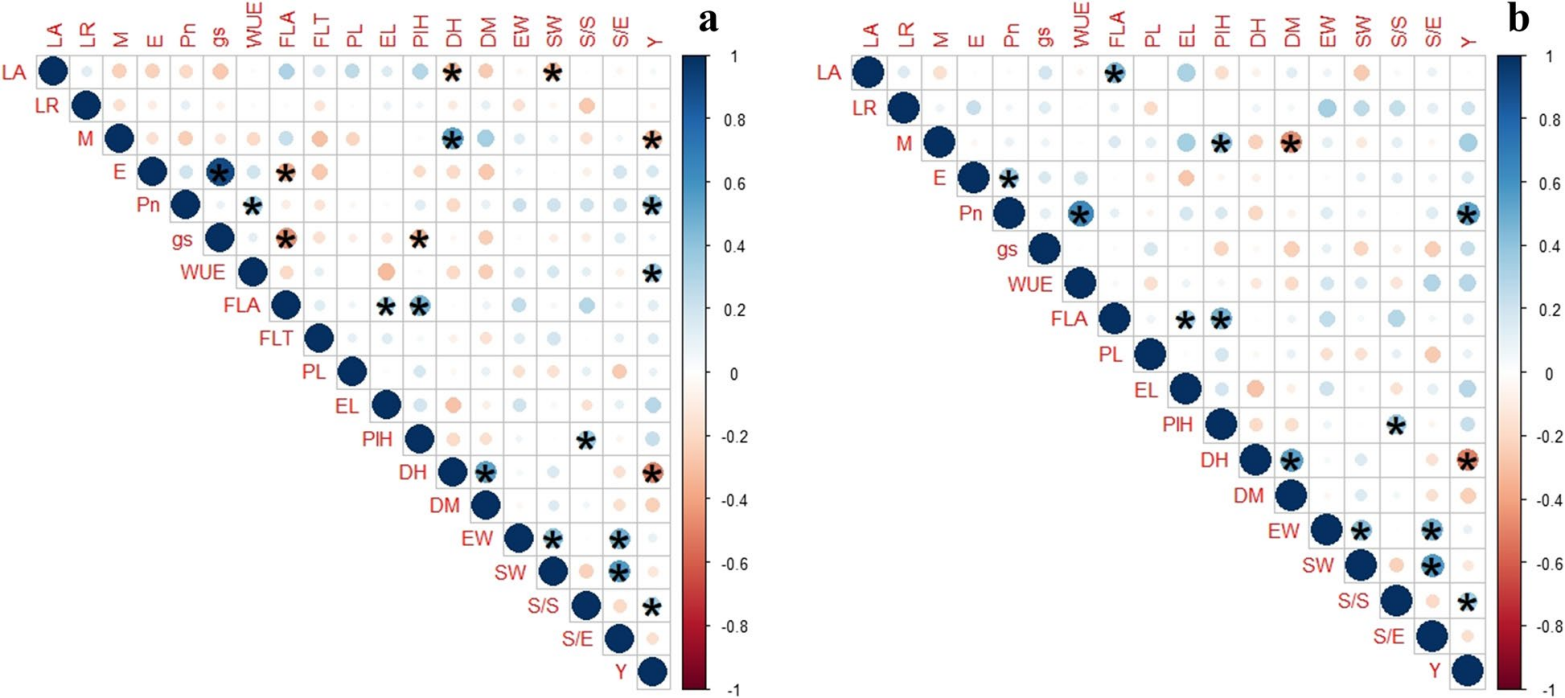
Phenotypic correlation for leaf traits (leaf angle and leaf rolling), soil moisture traits (moisture content in the root zone and vicinity and difference between both), physiological and yield traits. **a** Correlation analysis at the booting stage. **b** Correlation analysis at the anthesis stage. LA: leaf-stem angle, LR: leaf rolling; RZ: moisture content in the root zone; V: soil moisture content in the vicinity; RZ-V: Difference of soil moisture content in the root zone and vicinity, E Transpiration, Pn: Photosynthesis, gs: Stomatal conductance, WUE: Photosynthetic water use efficiency; FLA: flag leaf attitude; FLT: flag leaf twist; Y: Plot Yield; EW: Ear weight (g); SW: Seed weight/ear (g); S/S: Spikelet/spike; S/E: Grains/spike. The bar on the left side of the plot shows the value of the coefficient. The blue color indicates a positive correlation while the red color indicates a negative relationship. The deepness of the color indicates the strength of the correlation

Correlation analysis indicated significant negative correlations among LA, SW (seed weight), RZ, Pn and EW, RZ-V, and Y. However, significant positive correlations were found among the yield traits (Fig. 5). Interestingly, significant negative associations were found among leaf angle, leaf rolling, and the contact angle values while leaf traits showed positive association with the stem flow water collected by the collectors (Fig. S5). A strongly positive association was found between LA and LR while negative among leaf traits and soil moisture difference at all the stages, but the association was not significant (Fig. 5a, b, Fig. S4).

**Fig. 6:**
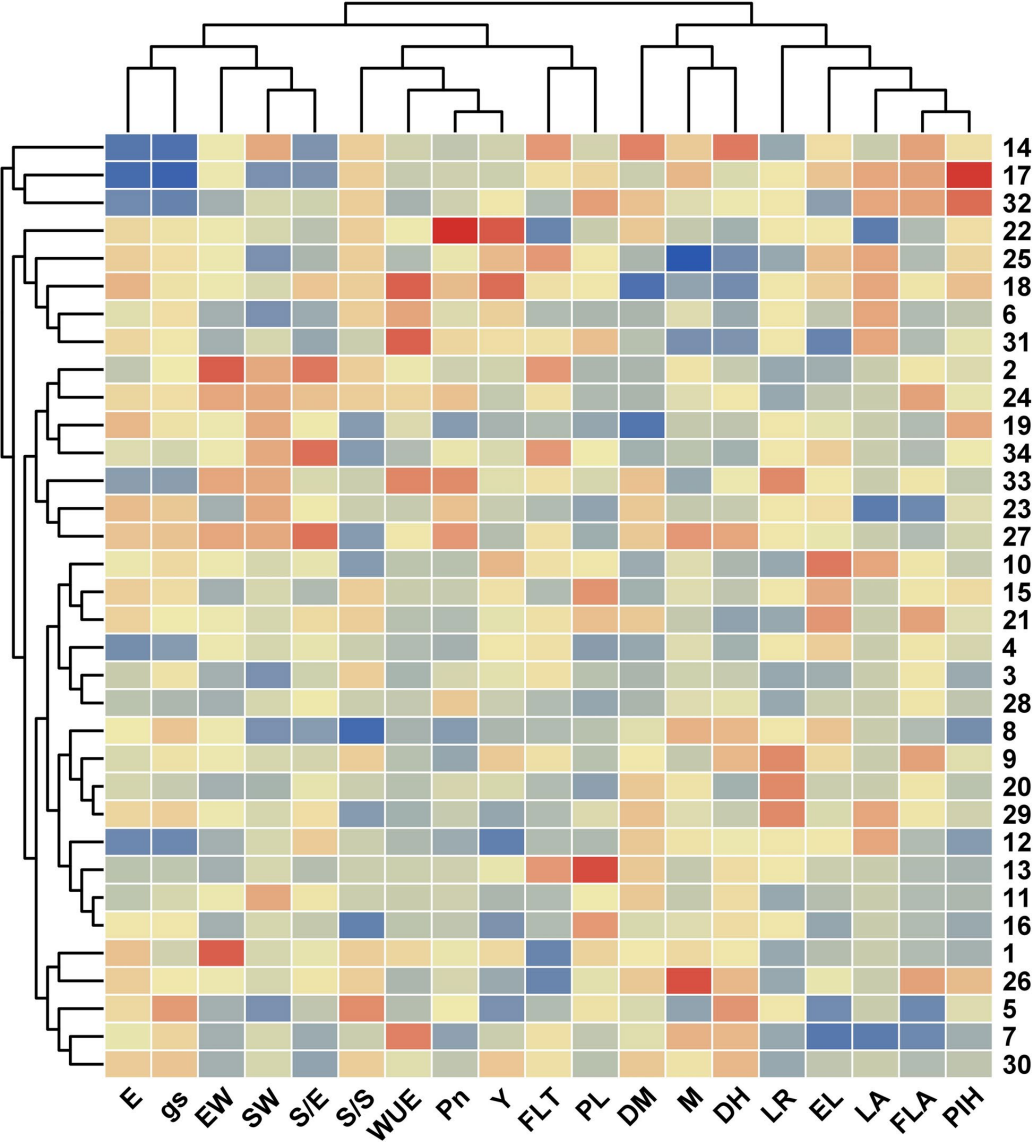
Heat map for morpho-physiological and yield traits for 34 wheat genotypes. LA: leaf angle; LR: leaf rolling; M: Difference of soil moisture content in the root zone and vicinity; E: Transpiration, Pn: Photosynthesis, gs: Stomatal conductance, WUE: Photosynthetic water use efficiency; EW: Ear weight; SW: seed weight; S/E: seeds per ear; DM: Days to maturity; DH: days to heading; PlH: plant height; FLA: flag leaf area; PL: peduncle length; EL: ear length; S/S: spikelet per spike; Y: plot yield

A heat map was developed for the selection of genotypes based on phenotypic combinations of all the traits under study as presented in Fig. 6. The heat map displayed variation among genotypes based on color. The intensity and variation of color represent the change in values being decreased gradually from blue to red i.e., the blue color indicates higher values, and the red color indicates lower values. The cluster-based heat map divided genotypes into ten phenotypic combinations. From each cluster, one representative genotype was selected as a core set for further experiments to study leaf wettability and stem flow.

### Stem flow and leaf wettability

**Fig. 7:**
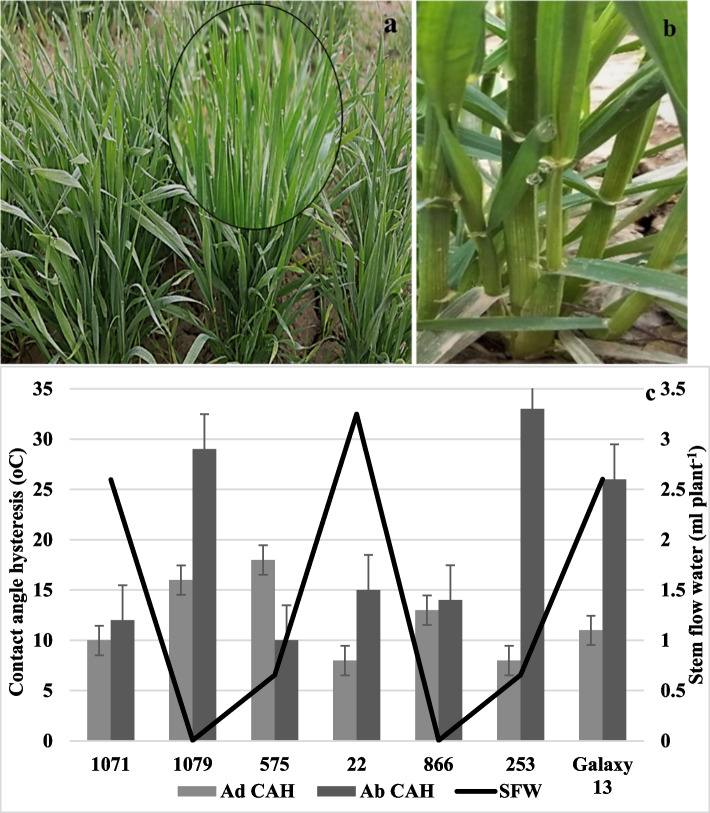
Stem flow mechanism of water channeling in wheat genotypes under natural field conditions. **a** Water captured by the wheat genotype in a plot under natural environment, the circle shows a zoom-in picture of the leaves bearing water droplets. **b** Movement of water droplets along the stem towards the base wetting the root zone. **c** Contact angle hysteresis of the abaxial and adaxial leaf surface, and stem flow water for the selected seven genotypes. CAH: contact angle hysteresis for abaxial (ab) and adaxial (ad) leaf surface, SFW: stem flow water

The fine water droplets captured by the leaf were seen to coalesce into droplets of larger size on the leaf edges (Fig. 7a). These droplets trickle down through the stem, reaching the ground (Fig. 7b), where soil dampness was evident and validated through soil moisture content. However, variation existed among genotypes for the stem flow capacity depending upon the leaf architecture (Fig. 7c). The correlation among the leaf traits, leaf surface wettability, and soil moisture content further validated these results (Fig. 6). The significant positive association among the stem flow water and wettability of the abaxial leaf surface while a significant negative association between adaxial leaf surface wettability, stem flow, and leaf angle indicated that genotypes with higher wettability of abaxial surface and lower wettability for adaxial surface were efficient in droplet channeling towards ground if they had erect leaf-stem angle (Fig. S4). Such properties were shown by genotype 7 which depicted maximum moisture harvested through the collector (3.25 ml) contrasting to genotypes 5 and 18 that captured the lowest water (0.00 ml) (Fig. 7c). Moreover, these genotypes also showed contrasting leaf angles i.e., genotype 7 being erect while genotypes 5 and 18 were semi-erect with inward leaf rolling (Fig. S3). However, genotypes with semi-droopy to droopy leaf angle (Galaxy-13) also showed significant fog water input due to the drip-ff mechanism from the droopy leaves. The CAH indicated that the genotypes with lower CAH values on both abaxial and adaxial surfaces (genotype 1 and 7) had higher stem flow water as compared to the genotypes with higher CAH values (genotype 5 and 18).

## Discussion

Breeding for climate change resilience is the main concern for the current research. Leaf functional traits play important role in drought and heat stress tolerance and utilization of air moisture as a valuable water source [25]. The exploitation of wheat genetic diversity for this purpose can be a targeted approach for climate-smart agriculture [26].

The leaf angle of cereals is associated with better light penetration, which consequently enhances photosynthesis and ultimately increases yield [16]. Similarly, leaf wettability and water retention are important leaf functional traits to enhance water availability in the root zone of the plants under arid and semi-arid conditions [27]. Therefore, the study focused to explore the genetic diversity of leaf angle, leaf wettability, and air moisture water retention as well as its movement along the stem towards the root zone. The analysis of variance showed significant variation among genotypes (Tables S1 and S2) favoring the hypothesis to explore genetic diversity of leaf traits for self-irrigation. It was found that the air moisture harvested through stem flow was remarkably higher (2.5–3.5 ml) for the genotypes with erect to semi-erect leaf angles and spiral leaf rolling (genotype 7) [28]. A wheat plant requires 2.6 mm water per day on an average calculated by keeping 400 mm water requirement by the wheat crop from germination to maturity [29]. The 1-3 ml of water per day obtained by a plant (Fig. 7c) with 5-10 tillers on an average corresponds to 10-14 mm water per day, so wheat can capture substantial amount of air moisture to fulfill its irrigation needs. As a result of water input through air moisture, the soil moisture increases near the plant bases especially for the plants with upright stature [9]. The fog collection occurs through the stem flow for the plants with erect habit, the water is conducted directly towards the rootzone. Soil moisture recorded near the root zone of the plant also indicated that moisture percentage in the root zone was 6-8% greater than the soil moisture in the vicinity for the genotypes with erect and moderately rolled leaves as compared to the droopy genotypes. However, the plants with droopy leaves showed heterogeneous soil moisture throughout the plot owing to fog drip from sagging leaves causing uneven water distribution within the plot. The improved water status after a fog event (measured by the soil moisture content) indicated that the water harvested is absorbed via root system of the plant. The amount of water collected in this study is enough evidence to claim wheat as an efficient moisture harvester. It corresponds well to the amount of water reported in other studies [19, 30–32]. Thus, such genotypes can perform better under drought stress conditions when other irrigation sources are not available [19]. C Holder [27] also supports that a steeper leaf angle increases the water channeling efficiency of the leaves. Interestingly, genotypes having erect leaf angle and inward to twist type rolling showed erect flag leaf and moderate flag leaf rolling as presented in Fig. 3a. It is also confirmed by the correlation analysis that genotypes with erect leaf angle were associated with the erect flag leaf attitude and spiral leaf rolling which can be helpful in air-moisture capturing [9]. The negative association of leaf traits with leaf wettability indicated that the erect stature and spiral rolling of genotypes was associated with the low contact angle values and high stem flow water (Fig. 5) [18].

The study showed that the surface wettability of leaves also influenced the air moisture harvesting efficiency by improving the water channeling property of the leaf [6]. The genotypes coded as 7 and 18, for example, had the lowest CAH comparatively, so these genotypes had the maximum amount of stem-flow water and soil moisture content in the root zone (Fig. 7). This can be explained by the fact these genotypes had comparatively erect to semi-erect leaf angle and hydrophilic leaf surface which helps in the beading of water droplets and give rise to stem flow [9].

For the selection of the best performing genotypes, genotype-trait biplot analysis was performed. The genotypes on the opposite side of the vector LA, FLA, and LR were considered best performing as the lowest score on the scale was desired for selection. For example, genotype 7 was found on the opposite side of the LA and FLA vector and in front of the M vector, indicating this genotype had the lowest value for LA and FLA (i.e., representing erect phenotype) while the maximum amount of soil moisture content (Fig. 4). But interestingly similar trend was found between the soil moisture difference and plot yield. So, the genotypes with low soil moisture content had higher plot yield (genotype18 and 22). The biplot also presents that the traits showed an almost similar trend of the association at both stages further verified by the correlation plot (Fig. 5). The flag leaf twist (FLT) showed an inverse relationship with the WUE, and Pn indicated that genotypes with the lowest flag leaf twist (< 30%) showed the highest values of photosynthesis (4.6 μmol CO_2_ m^− 2^ s^− 1^) and water use efficiency (3.7 mmol CO_2_ mol^− 1^ H_2_O) due to exposed leaf surface area. The genotypes 5, 17, 18 and 22, 28, 33 showed contrasting scores for the FLT, WUE and Pn i.e., higher values for FLT and lower values for Pn and WUE. So, these genotypes had a comparatively higher plot yield (Fig. S6). Similar has been reported in rice that variation in flag leaf attitude can be an important trait for yield enhancement in cereals [33].

The correlation plots indicated a significant positive relationship between the leaf angle and leaf wettability of the abaxial and adaxial surfaces. It means that contact angle values increase with the increasing leaf angle i.e., erect the leaf angle (<30^o^), more the surface will be hydrophobic (>90^o^ contact angle). Interestingly, the stem flow water showed a positive correlation with leaf wettability on the abaxial side while a negative correlation with the adaxial surface. It indicates that the stem flow water increases when the adaxial leaf surface of the wheat leaf is hydrophilic [34, 35]. Also, the alternate leaf surface properties on both sides increase the water input in the root zone by alternate droplet retention due to the hydrophilic property of the adaxial surface and increase droplet movement due to the hydrophobic property of the abaxial surface. Similar results have been supported by JM Wigzell, RC Racovita, BG Stentiford, M Wilson, MT Harris, IW Fletcher, DPK Mosquin, D Justice, SK Beaumont, R Jetter, et al. [6]. While the negative association between the leaf angle and seed weight (Fig. 5a) shows that the genotypes with erect leaf angle had higher seed weight per ear. Also, soil moisture content showed a significant negative correlation with the rate of photosynthesis indicating that higher soil moisture content lowers the rate of photosynthesis (Fig. 5a). That explains the fact the genotypes with higher soil moisture content (Genotype 7) had comparatively lower yields.

Concluding, the study reveals the mechanism and association of leaf hydrophilicity, stem flow, and leaf angle that play a key role in droplet retention and water movement. Both the surface structure and the leaf hydrophilicity as well as the minimum contact angle hysteresis enhanced the droplet coalescence as well as the effective transport of water towards the plant base. Therefore, this study paves the way to focus on finding nature-based solutions to develop climate resilience in economically important crops.

## Conclusions

Limited studies have been done in major crops to utilize air moisture as a potential irrigation water source. This study conducted a series of experiments including characterization of wheat genotypes under normal field conditions for leaf traits, screening of best genotypes, and their evaluation under field conditions through surface wettability traits and stem flow. Climate parameters were also observed and recorded during the whole growing season. It was revealed that enough air moisture was present during the critical growth stages of the wheat plant. The air moisture was seen condensed on the leaves of the wheat plant to a varying extent depending upon the plant architecture in the foggy as well as non-foggy days. This study found that the genotypes with an assembly of upright stature of the plant and, inward or spiral leaf rolling, leaf wettability, and droplet movement efficiency (low contact angle hysteresis) make up an efficient system for interception and utilization of air-borne moisture in wheat. These findings will be helpful for a wheat breeder to exploit for the development of drought-tolerant germplasm and can serve as a model for other cereal crops. However, the anatomical parameters of leaf surface like prickle hairs, epicuticular wax and arrangement of bulliform cells are directly associated to leaf parameters especially leaf wettability and leaf angle and would further be explored.

## Supplementary Information

**Additional file 1: Figure S1.** Historic climatic data of study area (Multan) from 1984 to 2018. **Figure S2.** Genotypic frequency distribution of 1796 wheat genotypes for leaf angle (a) and leaf rolling (b). **Table S1.** Distribution of 1796 wheat genotypes in various phenotypic combinations of four novel traits. **Figure S3.** The genotypes with dynamics of leaf angle growing under normal field conditions. **Figure S4.** Genotypic performance of the 34 wheat genotypes for leaf triats and physiological traits at the anthesis stage. a. Biplot analysis b. Correlation analysis. LA: leaf angle, LR: leaf rolling, M: Difference of soil moisture content in the root zone and vicinity, T: Transpiration, P: Photosynthesis, SC: Stomatal conductance, WUE: Photosynthetic water use efficiency. **Figure S5.** Association between the leaf traits (leaf angle and leaf rolling), surface wettability (adaxial and abaxial surface), soil moisture content and stem flow water. The bar on the left side of the plot shows the value of the coefficient. The blue color indicates a positive correlation while the red color indicates a negative relationship. The deepness of the color indicates the strength of the correlation. The cross in the bubble indicates a significant correlation. LA: leaf angle; LR: leaf rolling, Ad: contact angle of the adaxial leaf surface; ab: contact angle of the abaxial leaf surface; SFW: stem-flow water. **Figure S6.** Genotype-trait biplot analysis of yield traits for 34 wheat genotypes. **Table S2.** Mean square values for the leaf traits, soil moisture, and physiological traits. **Table S3.** Mean square values for the morphological and yield traits.

## Data Availability

All data generated or analyzed during this study are included in this published article [and its supplementary information files] and may also be requested from the corresponding authors.

## Abbreviations

LA: Leaf-stem angle
LR: Leaf rolling
M: Difference of soil moisture content in the root zone from vicinity
E: Transpiration
Pn: Photosynthesis
gs: Stomatal conductance
WUE: Photosynthetic water use efficiency
FLA: Flag leaf attitude
FLT: Flag leaf twist
RZ: Soil moisture content in the root zone
V: Soil moisture content in the vicinity
RZ-V: Difference of soil moisture content in the root zone from vicinity
Y: Plot yield
EW: Ear weight (g)
SW: Seed weight/ear (g)
S/S: Spikelet/spike
S/E: Grains/spike
DM: Days to maturity
DH: Days to heading
PlH: Plant height
FLA: Flag leaf area
PL: Peduncle length
EL: Ear length
ACA: Advancing contact angle
RCA: Receding contact angle
CAH: Contact angle hysteresis
SFW: Stem flow water
ab: Abaxial leaf surface
ad: Adaxial leaf surface
