# Supplementary material for: Leaf wettability and leaf angle affect air-moisture deposition in wheat for self-irrigation
Source: BMC Plant Biol. 2023 Feb 27;23:115. doi: 10.1186/s12870-023-04123-z (PMC9969695; doi:10.1186/s12870-023-04123-z)
Supplement: Supplementary file 1 — Additional file 1: Figure S1. Historic climatic data of study area (Multan) from 1984 to 2018. Figure S2. Genotypic frequency distribution of 1796 wheat genotypes for leaf angle (a) and leaf rolling (b). Table S1. Distribution of 1796 wheat genotypes in various phenotypic combinations of four novel traits. Figure S3. The genotypes with dynamics of leaf angle growing under normal field conditions. Figure S4. Genotypic performance of the 34 wheat genotypes for leaf triats and physiological traits at the anthesis stage. a. Biplot analysis b. Correlation analysis. LA: leaf angle, LR: leaf rolling, M: Difference of soil moisture content in the root zone and vicinity, T: Transpiration, P: Photosynthesis, SC: Stomatal conductance, WUE: Photosynthetic water use efficiency. Figure S5. Association between the leaf traits (leaf angle and leaf rolling), surface wettability (adaxial and abaxial surface), soil moisture content and stem flow water. The bar on the left side of the plot shows the value of the coefficient. The blue color indicates a positive correlation while the red color indicates a negative relationship. The deepness of the color indicates the strength of the correlation. The cross in the bubble indicates a significant correlation. LA: leaf angle; LR: leaf rolling, Ad: contact angle of the adaxial leaf surface; ab: contact angle of the abaxial leaf surface; SFW: stem-flow water. Figure S6. Genotype-trait biplot analysis of yield traits for 34 wheat genotypes. Table S2. Mean square values for the leaf traits, soil moisture, and physiological traits. Table S3. Mean square values for the morphological and yield traits. [file 12870_2023_4123_MOESM1_ESM.docx]

**Leaf wettability and leaf angle affect air-moisture deposition in wheat for self-irrigation**

Sadia Hakeem^1^, Zulfiqar Ali^1,4^,*, Muhammad Abu Bakar Saddique^1^, Sabah Merrium^1^, Muhammad Arslan^3^,^*^, Muhammad Habib-ur-Rahman^2^, ^3^,^*^

^1^Institute of Plant Breeding and Biotechnology, MNS University of Agriculture, Multan, Pakistan

^2^Department of Agronomy, MNS University of Agriculture, Multan, Pakistan

^3^Institute of Crop Science and Resource Conservation (INRES), Crop Science Group, University of Bonn, Germany

^4^Department of Plant Breeding and Genetics, University of Agriculture, Faisalabad, Pakistan

* Corresponding Email: [Zulfiqar_ali@uaf.edu.pk](mailto:Zulfiqar_ali@uaf.edu.pk): [mhabibur@uni-bonn.de](mailto:mhabibur@uni-bonn.de): [m.arslan@uni-bonn.de](mailto:m.arslan@uni-bonn.de)

**Supplementary Information**

**Figure S1**: Historic climatic data of study area (Multan) from 1984-2018


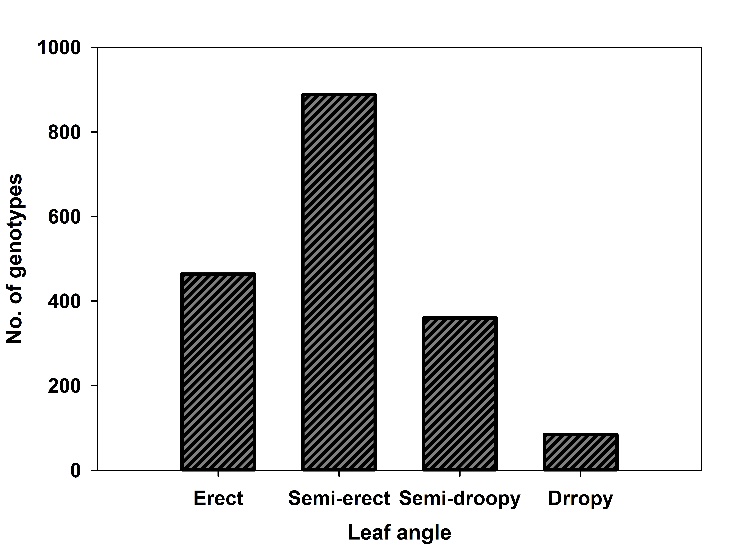

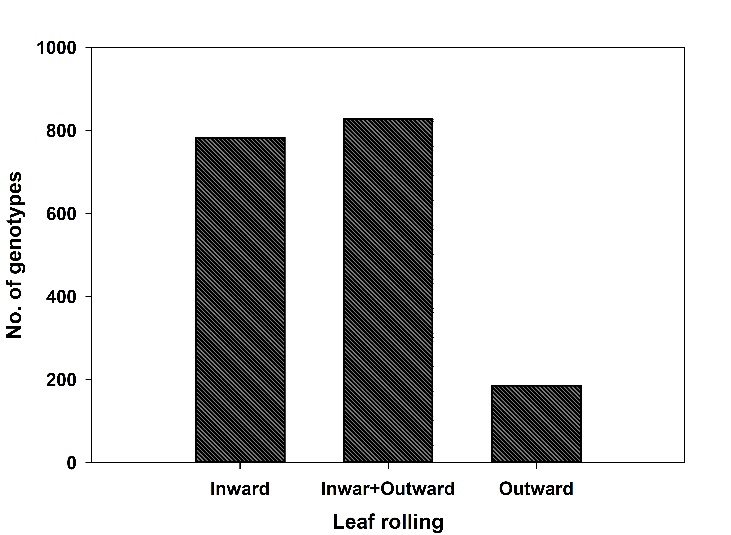


**b**

**a**

**Figure S2:** Genotypic frequency distribution of 1796 wheat genotypes for leaf angle (a) and leaf rolling (b)

**Table S1. Distribution of 1796 wheat genotypes in various phenotypic combinations of four novel traits.**

| **Leaf Rolling (LR)** | **Leaf Erectness (LE)** | **Groove types (GT)** | **Dense on adaxial, abaxial and edges of leaf** | **Light on adaxial, abaxial and edges of leaf** | **Dense on adaxial and abaxial surfaces of leaf** | **Light on adaxial and abaxial surfaces of leaf** | **Dense on abaxial and edges of leaf** | **Light on abaxial and edges of leaf** | **Dense on adaxial and edges of leaf** | **Light on adaxial and edges of leaf** | **Dense on abaxial surface of leaf** | **Light on abaxial surface of leaf** | **Dense on adaxial surface of leaf** | **Light on adaxial surface of leaf** | **Dense on edges of leaf** | **Light on edges of leaf** | **No prickle hairs** | **Total** |
| --- | --- | --- | --- | --- | --- | --- | --- | --- | --- | --- | --- | --- | --- | --- | --- | --- | --- | --- |
| Inward | Erect | Deep | 3 | 3 | 2 |  |  |  | 7 | 6 | 1 | 1 | 1 | 2 | 2 | 12 | **4** | 44 |
|  |  | Medium | 3 | 1 | 1 | 4 | 3 | 2 | 14 | 11 | 2 | 3 | 2 | 5 | 1 | 14 | 8 | 74 |
|  |  | Light |  | 1 | 1 | 1 | 1 |  | 8 | 14 |  | 1 |  | 5 | 6 | 14 | 7 | 59 |
|  | Semi-erect | Deep | 3 | 6 |  | 4 | 4 |  | 22 | 5 | 3 | 2 | 2 | 2 | 1 | 11 | 12 | 77 |
|  |  | Medium | 5 | 3 | 2 | 3 | 4 | 1 | 36 | 23 | 3 | 3 | 7 | 14 | 13 | 32 | 21 | 170 |
|  |  | Light |  | 4 | 3 | 3 |  | 2 | 20 | 22 | 2 | 1 | 4 | 12 | 19 | 30 | 21 | 143 |
|  | Semi-droopy | Deep |  |  | 1 |  |  |  | 6 | 1 | 1 |  |  | 1 | 3 | 8 | 4 | 25 |
|  |  | Medium | 2 | 1 |  | 2 | 1 | 1 | 8 | 10 | 1 |  | 1 | 3 | 7 | 16 | 8 | 61 |
|  |  | Light |  |  | 2 | 1 |  | 1 | 9 | 15 | 2 | 1 |  | 7 | 4 | 15 | 16 | 73 |
|  | Droopy | Deep |  |  |  | 1 |  |  |  |  |  |  |  | 3 | 1 | 2 | 3 | 10 |
|  |  | Medium |  |  |  |  |  |  |  | 2 |  | 1 |  |  | 6 | 3 | 12 | 24 |
|  |  | Light | 1 |  |  |  |  |  | 4 | 4 |  |  |  | 2 | 3 | 6 | 3 | 23 |
| In+outward | Erect | Deep |  | 2 | 1 | 2 | 1 | 2 | 2 | 3 | 3 | 2 | 1 | 1 | 3 | 5 | 11 | 39 |
|  |  | Medium |  | 3 | 5 | 2 | 1 | 3 | 12 | 11 | 3 | 2 | 7 | 10 | 1 | 32 | 29 | 121 |
|  |  | Light | 1 |  | 1 | 3 | 1 |  | 5 | 9 | 3 | 5 | 2 | 11 | 1 | 16 | 20 | 78 |
|  | Semi-erect | Deep | 3 | 3 | 8 | 6 | 3 | 4 | 12 | 13 | 5 | 6 | 7 | 5 | 3 | 8 | 11 | 97 |
|  |  | Medium | 3 | 4 | 10 | 15 | 4 | 8 | 24 | 14 | 6 | 5 | 11 | 17 | 1 | 37 | 34 | 193 |
|  |  | Light |  | 2 | 9 | 2 | 1 | 5 | 13 | 6 | 4 | 3 | 7 | 17 | 5 | 25 | 30 | 129 |
|  | Semi-droopy | Deep |  | 1 |  | 1 | 2 | 1 | 2 |  | 2 |  | 2 | 1 | 1 | 5 | 4 | 22 |
|  |  | Medium | 2 | 2 | 2 |  |  | 1 | 5 | 4 | 2 |  | 1 | 11 | 2 | 17 | 11 | 60 |
|  |  | Light | 1 | 1 | 2 | 2 | 3 | 3 | 4 | 4 | 1 | 1 |  | 3 | 2 | 25 | 17 | 69 |
|  | Droopy | Deep |  |  |  |  |  | 1 | 2 |  |  |  |  |  |  |  | 1 | 4 |
|  |  | Medium |  |  | 1 |  |  |  |  |  | 1 |  | 2 | 1 |  | 2 | 2 | 9 |
|  |  | Light |  |  |  |  |  | 1 |  |  |  | 1 |  |  |  | 1 | 2 | 5 |
| Outward | Erect | Deep |  | 1 |  |  | 2 | 1 | 2 |  | 1 |  | 1 | 1 |  |  | 1 | 10 |
|  |  | Medium | 1 | 2 |  | 2 | 3 | 2 | 1 | 1 | 1 | 1 |  | 3 | 1 | 2 | 2 | 22 |
|  |  | Light | 1 |  | 2 | 2 |  |  | 2 |  | 1 |  | 2 | 1 |  | 4 | 2 | 17 |
|  | Semi-erect | Deep | 2 | 1 | 1 | 2 |  | 1 | 3 | 3 | 3 |  | 2 | 1 |  | 5 | 4 | 28 |
|  |  | Medium |  | 2 | 4 | 2 | 1 |  | 5 | 1 | 1 |  | 1 | 1 | 1 | 1 | 3 | 23 |
|  |  | Light | 1 | 6 | 3 | 1 | 1 | 1 | 2 | 2 | 2 |  | 1 | 1 | 4 | 2 | 1 | 28 |
|  | Semi-droopy | Deep | 1 | 1 |  |  |  |  |  |  |  |  |  |  |  |  | 1 | 3 |
|  |  | Medium | 2 | 5 |  | 2 | 1 |  | 1 | 4 |  |  | 2 | 1 | 3 | 4 | 2 | 27 |
|  |  | Light | 2 | 2 | 1 | 1 |  | 1 |  | 1 |  |  |  | 1 | 1 | 8 | 2 | 20 |
|  | Droopy | Deep |  |  |  |  |  |  |  |  |  |  |  |  | 1 |  |  | 1 |
|  |  | Medium |  |  |  | 1 |  |  |  |  | 1 |  |  |  |  | 1 |  | 3 |
|  |  | Light | 2 |  | 1 |  |  | 1 |  |  |  |  |  |  |  |  | 1 | 5 |
| Total |  |  | 39 | 57 | 63 | 65 | 37 | 43 | 231 | 189 | 55 | 39 | 66 | 143 | 96 | 363 | 310 | 1796 |


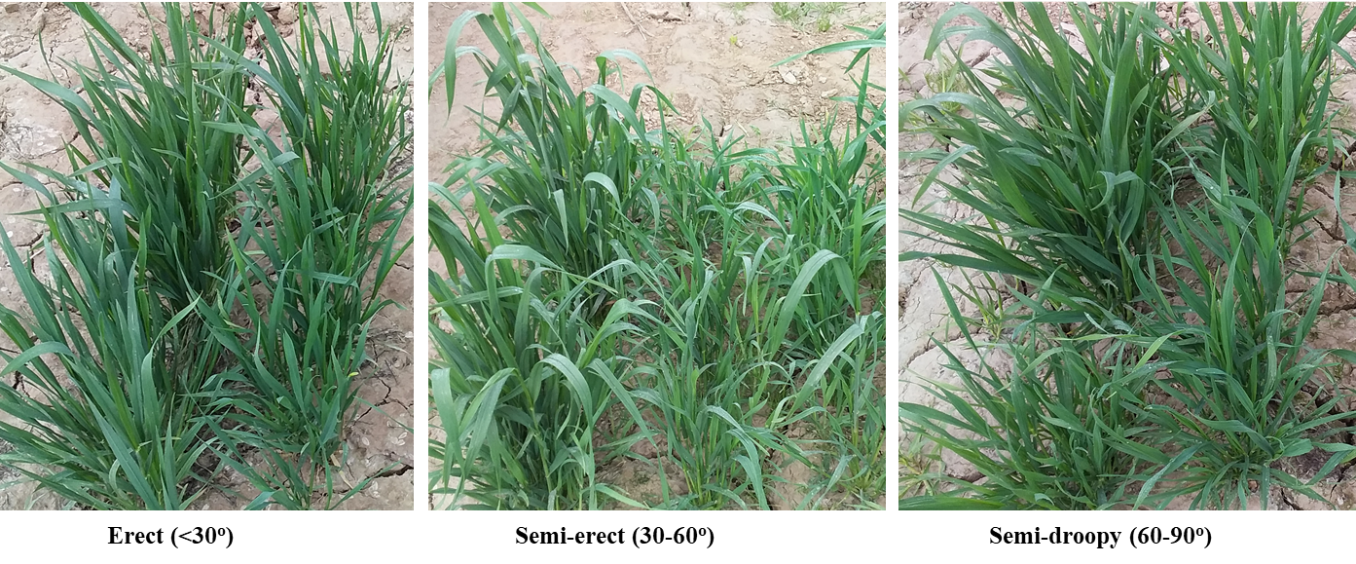


**Figure S3:** The genotypes with dynamics of leaf angle growing under normal field conditions


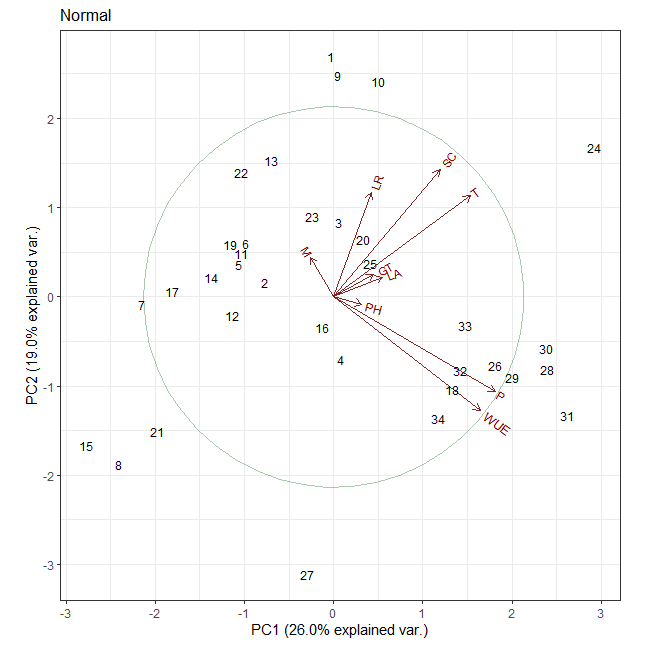

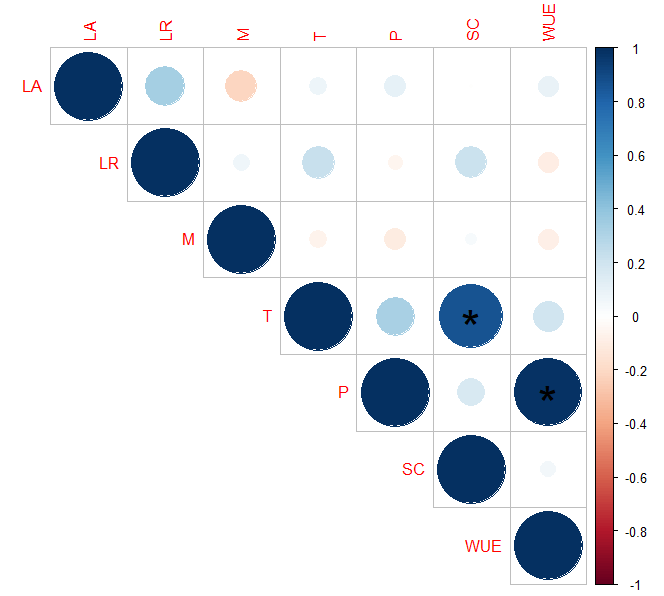


**Figure S4:** Genotypic performance of the thirty four wheat genotypes for leaf triats and physiological traits at the anthesis stage. a. Biplot analysis b. Correlation analysis. LA: leaf angle, LR: leaf rolling, M: Difference of soil moisture content in the root zone and vicinity, T: Transpiration, P: Photosynthesis, SC: Stomatal conductance, WUE: Photosynthetic water use efficiency


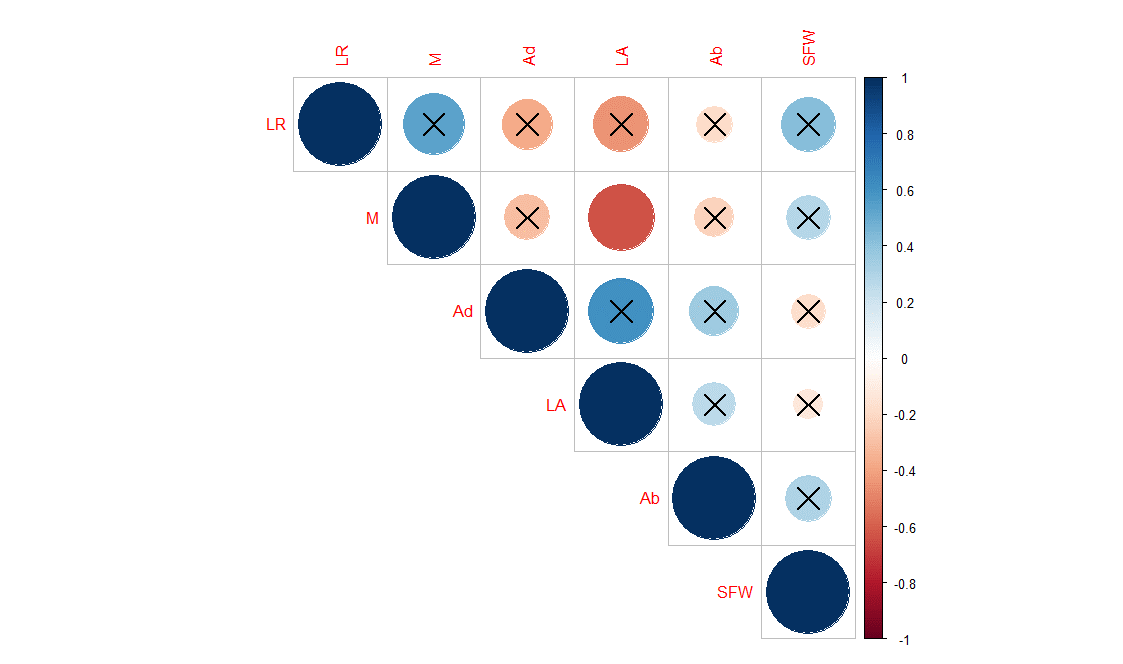


**Figure S5:** Association between the leaf traits (leaf angle and leaf rolling), surface wettability (adaxial and abaxial surface), soil moisture content and stem flow water. The bar on the left side of the plot shows the value of the coefficient. The blue color indicates a positive correlation while the red color indicates a negative relationship. The deepness of the color indicates the strength of the correlation. The cross in the bubble indicates a significant correlation. LA: leaf angle; LR: leaf rolling, Ad: contact angle of the adaxial leaf surface; ab: contact angle of the abaxial leaf surface; SFW: stem-flow water.


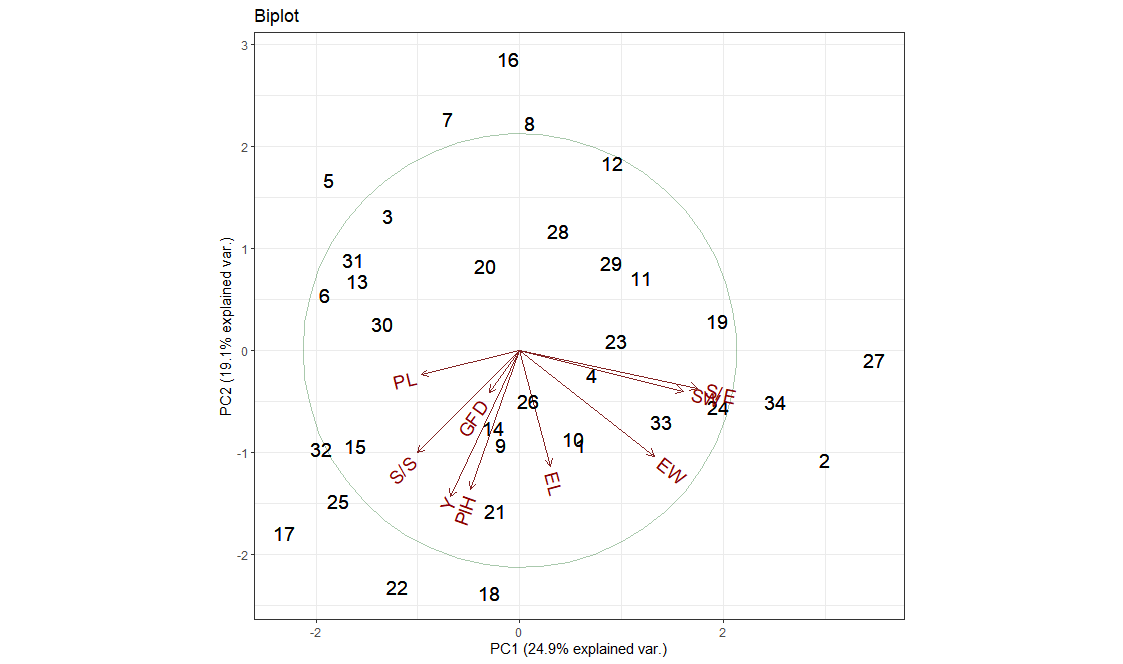


**Figure S6:** Genotype-trait biplot analysis of yield traits for thirty-four wheat genotypes

**Table S2: Mean square values for the leaf traits, soil moisture, and physiological traits**

| **SOV** | **Df** | **LA** | **LR** | **M** | **T** | **P** | **gs** | **WUE** |
| --- | --- | --- | --- | --- | --- | --- | --- | --- |
| Replication | 1 | 0.0 | 0.53. | 1.01** | 39.35*** | 3.2 | 0.0 | 0.4 |
| Genotype | 33 | 0.32487** | 0.68*** | 2.71*** | 0.1 | 2.94. | 805.5 | 8.32*** |
| Error | 33 | 0.3 | 0.1 | 0.1 | 0.2 | 1.8 | 7.2 | 0.1 |

LA: leaf angle; LR: leaf rolling; M: soil moisture content; T: transpiration rate; P: photosynthesis, gs: stomatal conductance; WUE: water use efficiency

**Table S3: Mean square values for the morphological and yield traits**

| **SOV** | **Df** | **FLA** | **FLT** | **PL** | **EL** | **PlH** | **GFD** | **EW** | **SW** | **S/S** | **S/E** | **Y** |
| --- | --- | --- | --- | --- | --- | --- | --- | --- | --- | --- | --- | --- |
| Replication | 1 | 0.1 | 0.1 | 4.0 | 1.4 | 44.5 | 2.88** | 1.7 | 0.9 | 3.3 | 18.0 | 408.5 |
| Genotype | 33 | 1.49*** | 1.15*** | 49.82** | 2.51*** | 285.70*** | 132.13*** | 1.34*** | 0.76*** | 6.88*** | 315.72*** | 6055.5*** |
| Error | 33 | 0.2 | 0.1 | 16.9 | 0.5 | 20.1 | 0.3 | 0.0 | 0.1 | 0.8 | 3.3 | 2144.7 |

FLA: flag leaf attitude; FLT: flag leaf twist; PL: peduncle length; PlH: plant height; GFD: grain filling duration; EW: ear weight; SW: seed weight; S/S: spikelets per spike; S/E: seeds per ear; Y: grain yield
